# Supplementary material for: Neural circuit mechanisms of hierarchical sequence learning tested on large-scale recording data
Source: PLoS Comput Biol. 2022 Jun 21;18(6):e1010214. doi: 10.1371/journal.pcbi.1010214 (PMC9249189; doi:10.1371/journal.pcbi.1010214)
Supplement: S1 Fig — Correlation coefficient between somatic and dendritic activity during learning in a task considered in Fig 2 is shown. Here, the 1,000 seconds-long learning period was divided into multiple training sections and the correlation coefficient between somatic and dendrite activity was calculated in each section. The solid line and shaded area (invisible) represent the mean and the s.d. of correlation over 10 independent simulations. (PDF) [file pcbi.1010214.s001.pdf]

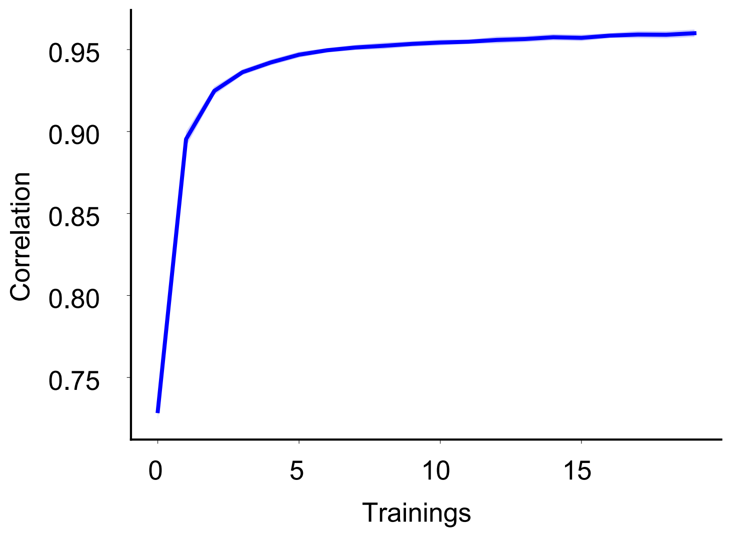

S1 Fig. Learning curve of recurrent gating network. Correlation coefficient between somatic and dendritic activity during learning in a task considered in Fig.2 is shown. Here, the 1,000 seconds-long learning period was divided into multiple training sections and the correlation coefficient between somatic and dendrite activity was calculated in each section. The solid line and shaded area (invisible) represent the mean and the s.d. of correlation over 10 independent simulations.
