# Supplementary material for: Neural circuit mechanisms of hierarchical sequence learning tested on large-scale recording data
Source: PLoS Comput Biol. 2022 Jun 21;18(6):e1010214. doi: 10.1371/journal.pcbi.1010214 (PMC9249189; doi:10.1371/journal.pcbi.1010214)
Supplement: S2 Fig — (a) Output spike trains of the trained recurrent network are shown. Neurons were sorted according to their onset response times. (b) The responses to the two chunks were averaged over 20 trials. (c) PCA was applied to obtain the low-dimensional trajectories of the trained network. The black, orange, and blue portions indicate the periods of random spike input, chunk 1 and chunk 2, respectively. The two trajectories corresponding to the two chunks were inseparable and the network failed to learn the chunks. (PDF) [file pcbi.1010214.s002.pdf]

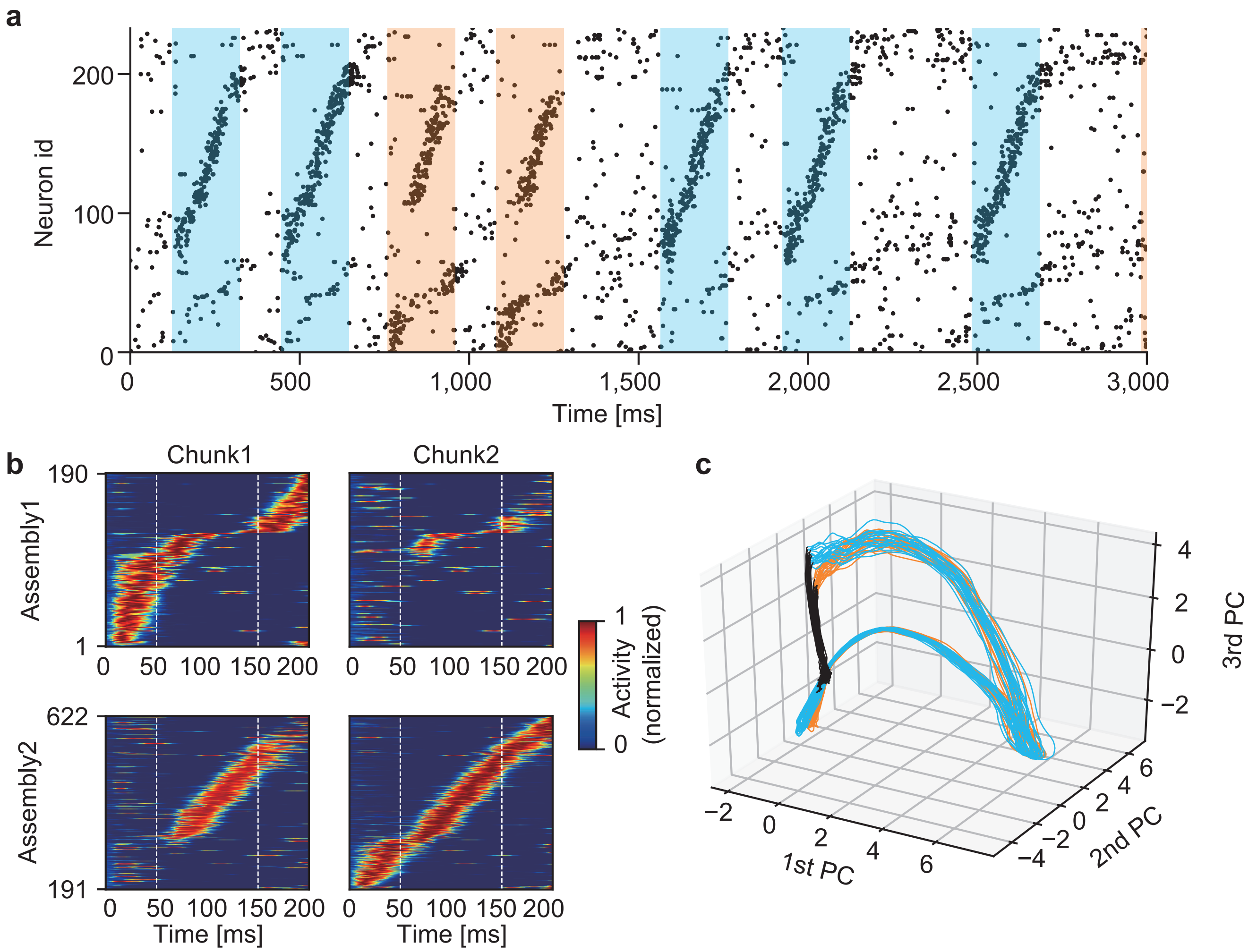

S2 Fig. Learning of overlapping patterns in a recurrent network without gating. (a) Output spike trains of the trained recurrent network are shown. Neurons were sorted according to their onset response times. (b) The responses to the two chunks were averaged over 20 trials. (c) PCA was applied to obtain the low-dimensional trajectories of the trained network. The black, orange, and blue portions indicate the periods of random spike input, chunk 1 and chunk 2, respectively. The two trajectories corresponding to the two chunks were inseparable and the network failed to learn the chunks.
