# Supplementary material for: Neural circuit mechanisms of hierarchical sequence learning tested on large-scale recording data
Source: PLoS Comput Biol. 2022 Jun 21;18(6):e1010214. doi: 10.1371/journal.pcbi.1010214 (PMC9249189; doi:10.1371/journal.pcbi.1010214)
Supplement: S3 Fig — (a) The responses of the first cell assembly to its preferred (left) and non-preferred (middle, right) chunks are shown. These responses were averaged and normalized as in Fig 2C. (b) Preferred responses of two neurons are shown as examples. Top and bottom traces show the responses of chunk2-D and chunk3-D selective neurons, respectively. (c) As in Fig 3C–3E, trial-averaged responses of dendrite, gating factor and soma are shown for two other neurons. (PDF) [file pcbi.1010214.s003.pdf]

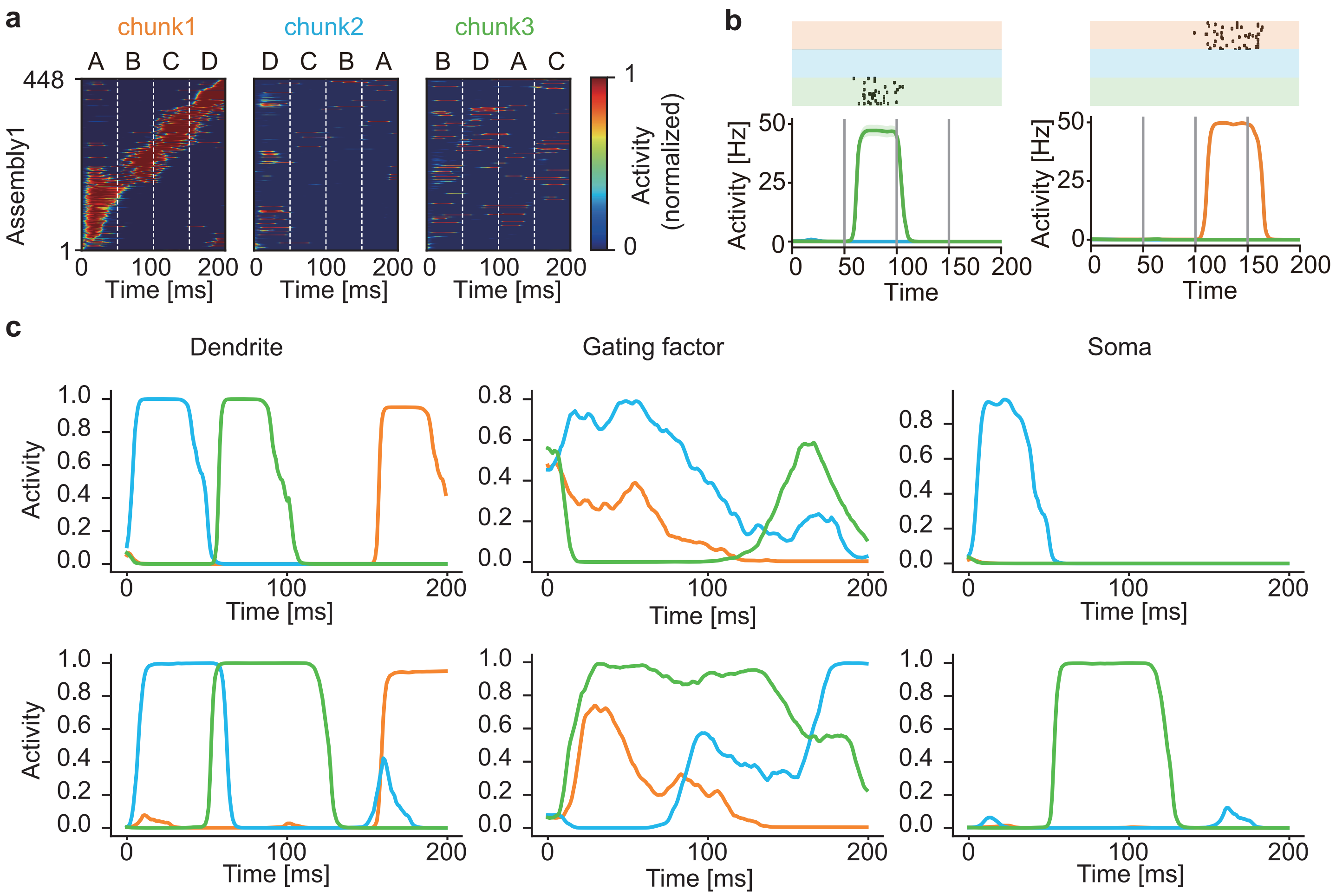

S3 Fig. Chunk-selective responses in the network trained in Fig. 3. (a) The responses of the first cell assembly to its preferred (left) and non-preferred (middle, right) chunks are shown. These responses were averaged and normalized as in Fig. 2c. (b) Preferred responses of two neurons are shown as examples. Top and bottom traces show the responses of chunk2-D and chunk3-D selective neurons, respectively. (c) As in Fig. 3c-e, trial-averaged responses of dendrite, gating factor and soma are shown for two other neurons.
