# Supplementary material for: Neural circuit mechanisms of hierarchical sequence learning tested on large-scale recording data
Source: PLoS Comput Biol. 2022 Jun 21;18(6):e1010214. doi: 10.1371/journal.pcbi.1010214 (PMC9249189; doi:10.1371/journal.pcbi.1010214)
Supplement: S4 Fig — (a) The average values of the weights between 12 assemblies defined according to the selective response to the 4 component patterns of the 3 chunks are shown. (b) Mean weights between groups of neurons in three cases are shown. Cyan and green colors indicate the connections project to assemblies correspond to previous and next component patterns within chunk, while magenta indicates interactions between assemblies correspond to the same component patterns but belong to different chunks. (PDF) [file pcbi.1010214.s004.pdf]

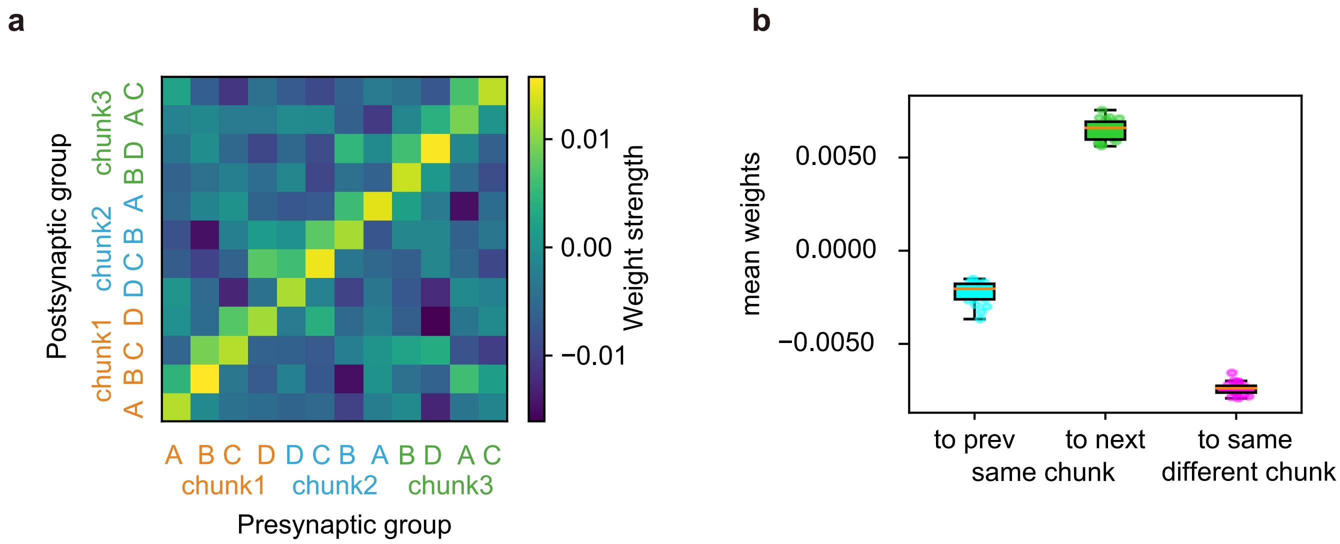

S4 Fig. Analysis of trained gating recurrent connections. (a) The average values of the weights between 12 assemblies defined according to the selective response to the 4 component patterns of the 3 chunks are shown. (b) Mean weights between groups of neurons in three cases are shown. Cyan and green colors indicate the connections project to assemblies correspond to previous and next component patterns within chunk, while magenta indicates interactions between assemblies correspond to the same component patterns but belong to different chunks.
