# Supplementary material for: Neural circuit mechanisms of hierarchical sequence learning tested on large-scale recording data
Source: PLoS Comput Biol. 2022 Jun 21;18(6):e1010214. doi: 10.1371/journal.pcbi.1010214 (PMC9249189; doi:10.1371/journal.pcbi.1010214)
Supplement: S5 Fig — (a) Performances of networks with sizes 600 and 300 are shown over different number of chunks are shown. Error bars show s.d.s. (b) Learning performances of network of size 600 over various values of parameter γ (see Eq (7) and (8)) are shown. Error bars show s.d.s. (c) Same as in (b), but over the strength of inhibition are shown. (PDF) [file pcbi.1010214.s005.pdf]

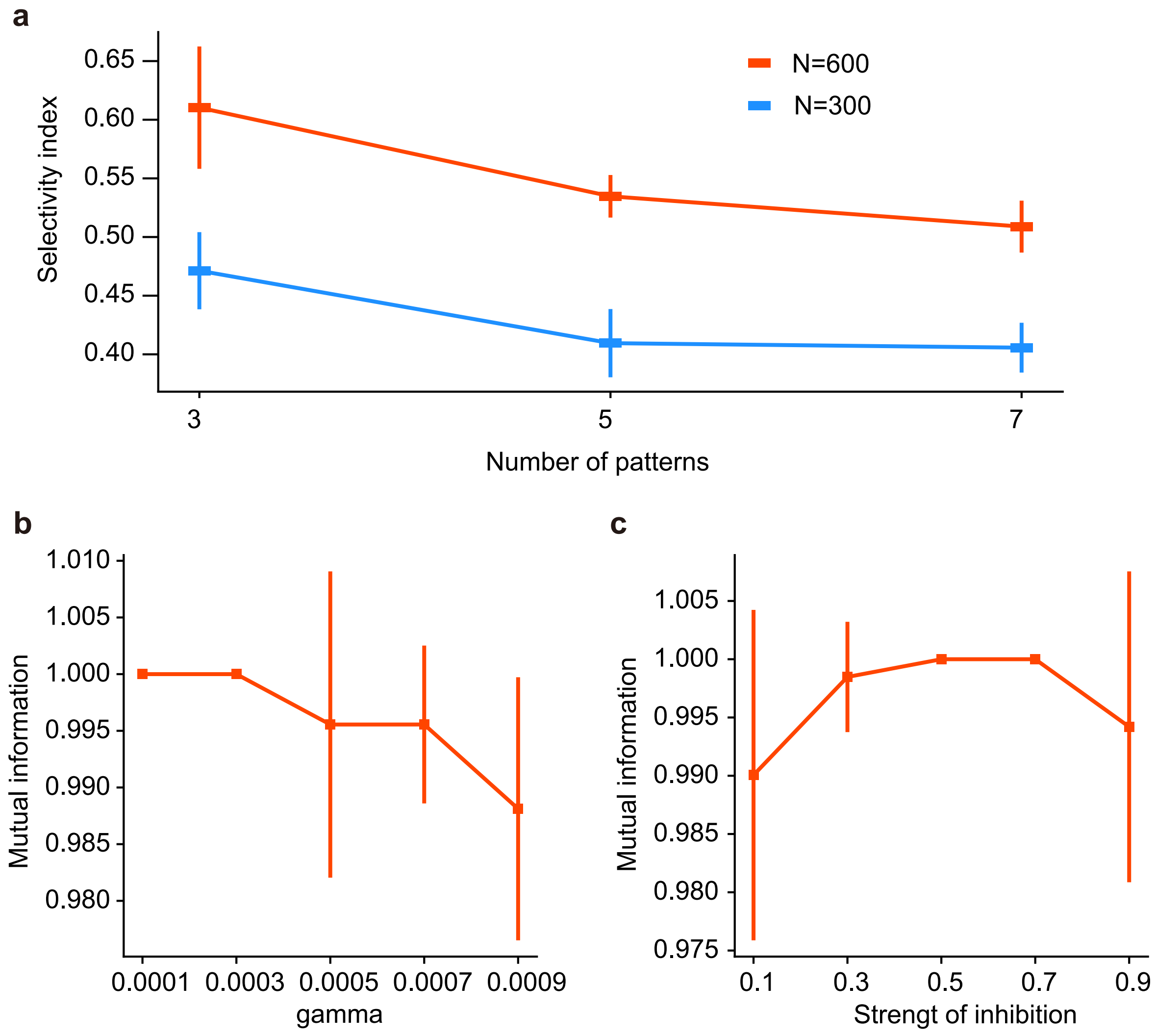

S5 Fig. Model performance dependences on various parameters. (a) Performances of networks with sizes 600 and 300 are shown over different number of chunks are shown. Error bars show s.d.s. (b) Learning performances of network of size 600 over various values of parameter γ (see Eq. (7) and (8)) are shown. Error bars show s.d.s. (c) Same as in (b), but over the strength of inhibition are shown.
