# Supplementary material for: Neural circuit mechanisms of hierarchical sequence learning tested on large-scale recording data
Source: PLoS Comput Biol. 2022 Jun 21;18(6):e1010214. doi: 10.1371/journal.pcbi.1010214 (PMC9249189; doi:10.1371/journal.pcbi.1010214)
Supplement: S6 Fig — (a) Responses of the networks trained on input spike trains with timing jitters of 70 ms (top) and 100 ms (bottom) are shown. Here, spike times within chunks were sifted by the amounts drawn by a Gaussian distribution with mean zero and s.d of jitter strength, and these jitters were present during learning and testing. Neurons were sorted according to the times of their response onsets during chunks, and only 160 out of the total 500 neurons are shown for the visualization purpose. (b) The normalized average activities of the two assemblies with timing jitters of 70 ms (left) and 100 ms (right) are shown. (c) Learning curves are shown when the average jitter was 0 ms (purple), 70 ms (green), and 100 ms (blue), respectively. The solid lines and shaded areas represent the averages and s.d over 20 trials, respectively. Learning performance was measured by the normalized mutual information between network activity and target labels (Methods). (d) The performance measures averaged over 20 trials are shown at various sizes of jitters. Error bars stand for the s.d. (PDF) [file pcbi.1010214.s006.pdf]

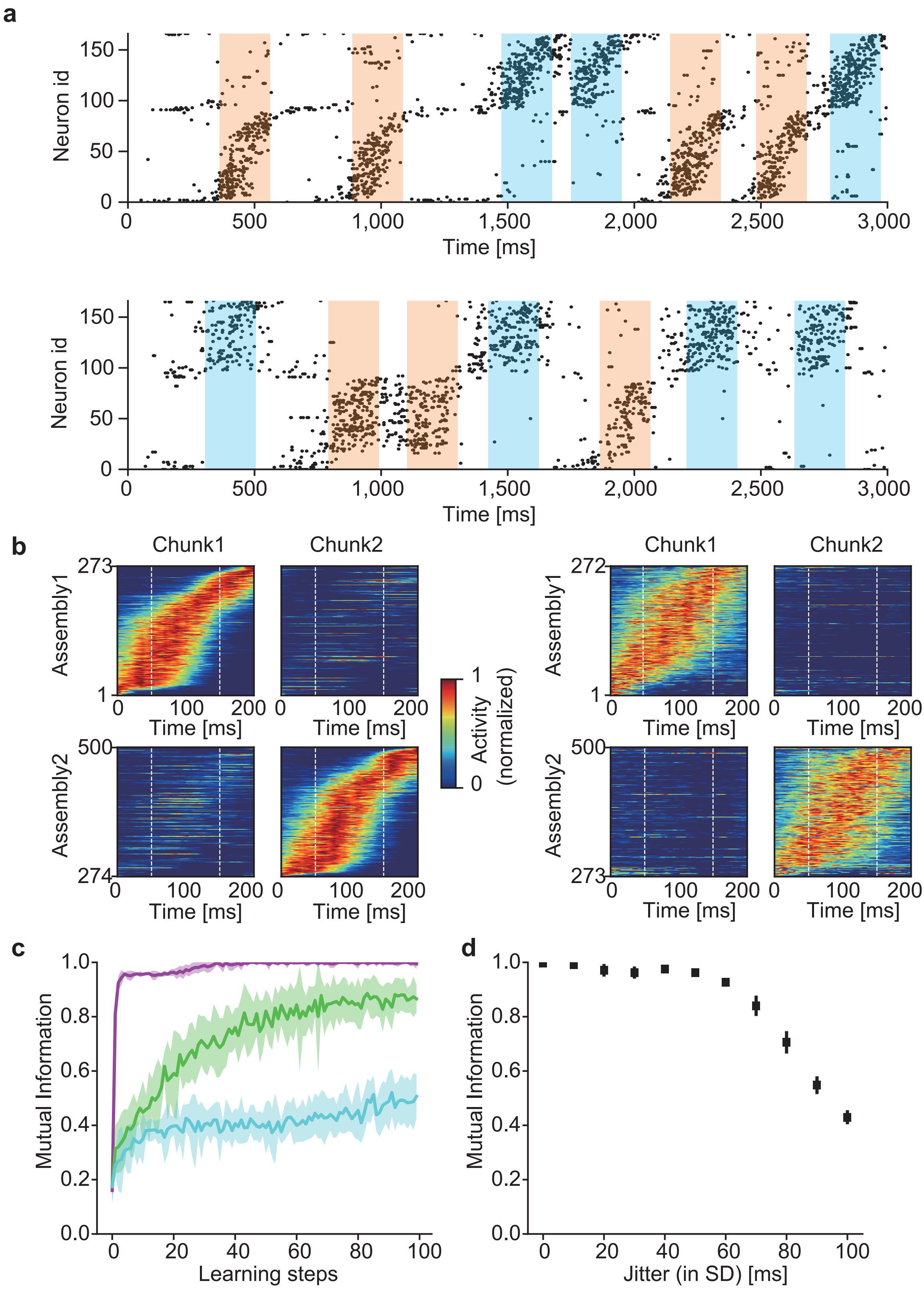

S6 Fig. Robustness against spike timing jitters. (a) Responses of the networks trained on input spike trains with timing jitters of 70 ms (top) and 100 ms (bottom) are shown. Here, spike times within chunks were sifted by the amounts drawn by a Gaussian distribution with mean zero and s.d of jitter strength, and these jitters were present during learning and testing. Neurons were sorted according to the times of their response onsets during chunks, and only 160 out of the total 500 neurons are shown for the visualization purpose. (b) The normalized average activities of the two assemblies with timing jitters of 70 ms (left) and 100 ms (right) are shown. (c) Learning curves are shown when the average jitter was 0 ms (purple), 70 ms (green), and 100 ms (blue), respectively. The solid lines and shaded areas represent the averages and s.d over 20 trials, respectively. Learning performance was measured by the normalized mutual information between network activity and target labels (Methods). (d) The performance measures averaged over 20 trials are shown at various sizes of jitters. Error bars stand for the s.d.
