# Supplementary material for: Neural circuit mechanisms of hierarchical sequence learning tested on large-scale recording data
Source: PLoS Comput Biol. 2022 Jun 21;18(6):e1010214. doi: 10.1371/journal.pcbi.1010214 (PMC9249189; doi:10.1371/journal.pcbi.1010214)
Supplement: S7 Fig — Running speed (top), whisking (middle), and pupil area (bottom) of freely behaving mouse are shown. (PDF) [file pcbi.1010214.s007.pdf]

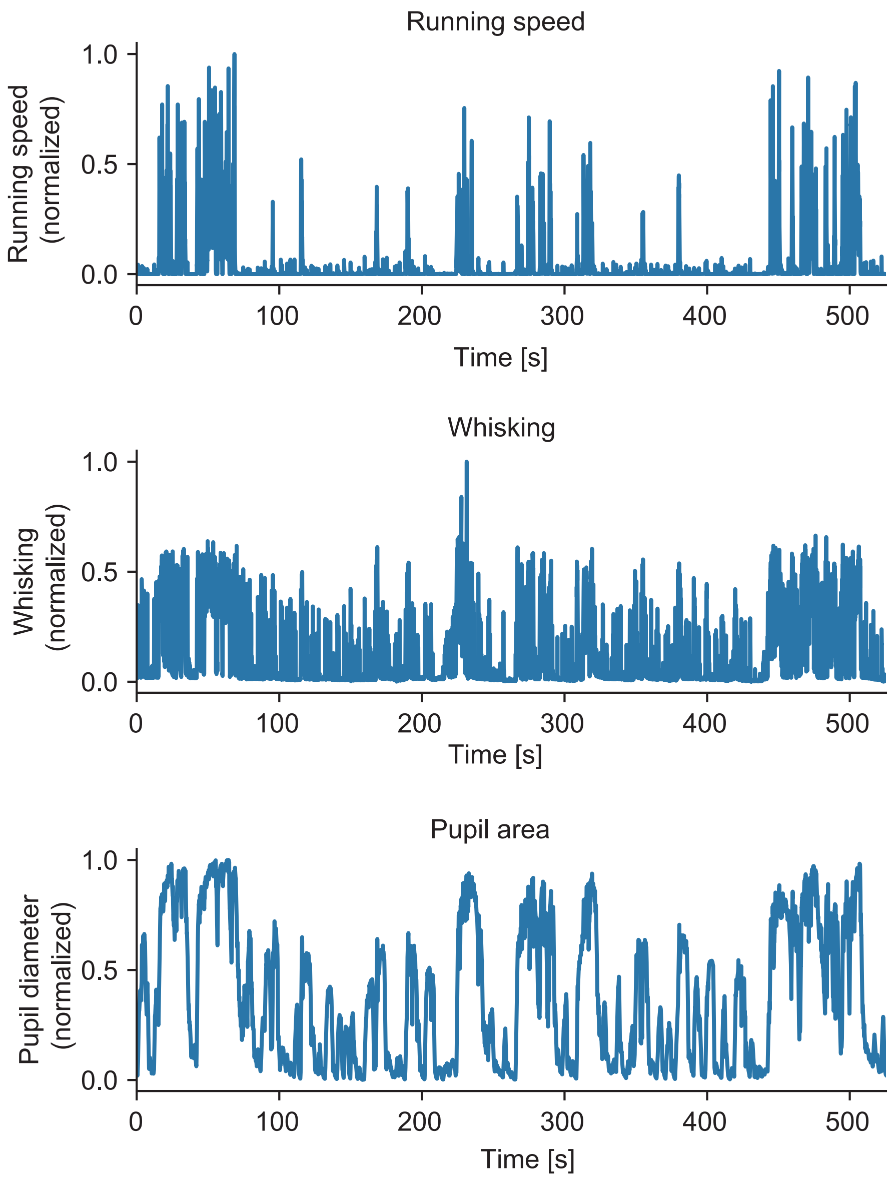

S7 Fig. Behaviors of freely behaving mouse. Running speed (top), whisking (middle), and pupil area (bottom) of freely behaving mouse are shown.
