# Supplementary material for: Neural circuit mechanisms of hierarchical sequence learning tested on large-scale recording data
Source: PLoS Comput Biol. 2022 Jun 21;18(6):e1010214. doi: 10.1371/journal.pcbi.1010214 (PMC9249189; doi:10.1371/journal.pcbi.1010214)
Supplement: S8 Fig — (a) The positions of a mouse on a linear track [23] (top) and the activities of model neurons learned without recurrent gating (bottom) are shown. Model neurons were sorted according to their onset response times. Separations between the two sequences corresponding to forward and backward runs are invisible (c.f. Fig 6B). (b) Activities of model neurons trained on the neural data recorded from the mice visual cortex [24,25] without recurrent gating are shown. The model neurons were sorted according to their onset response times. (c) We associated each cortical neuron with a model neuron having the highest correlation with the cortical neuron. Then, we sorted the cortical neurons according to the serial order of model neurons shown in (b). (d) Population-averaged activities of network model trained on the data of visual cortex with (left) and without (right) gating are shown. (e) Correlation between average activities shown in (d) and various behaviors are shown. Blue and magenta plots correspond to gating and non-gating, respectively. In both type of networks, 10 independent simulations were performed. (PDF) [file pcbi.1010214.s008.pdf]

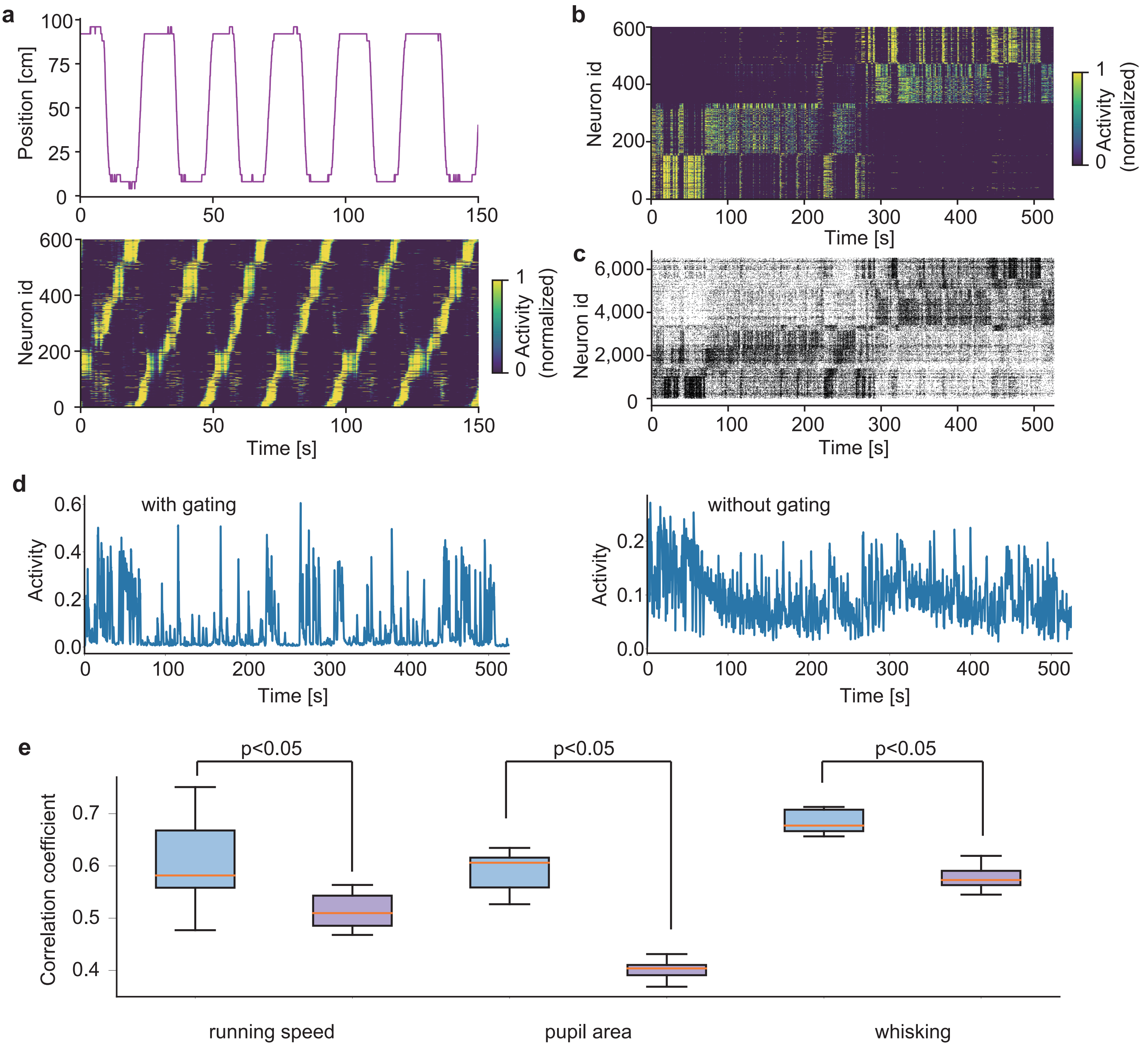

S8 Fig. Analysis of calcium imaging data without recurrent gating. (a) The positions of a mouse on a linear track [23] (top) and the activities of model neurons learned without recurrent gating (bottom) are shown. Model neurons were sorted according to their onset response times. Separations between the two sequences corresponding to forward and backward runs are invisible (c.f. Fig. 6b). (b) Activities of model neurons trained on the neural data recorded from the mice visual cortex [24,25] without recurrent gating are shown. The model neurons were sorted according to their onset response times. (c) We associated each cortical neuron with a model neuron having the highest correlation with the cortical neuron. Then, we sorted the cortical neurons according to the serial order of model neurons shown in (b). (d) Population-averaged activities of network model trained on the data of visual cor-tex with (left) and without (right) gating are shown. (e) Correlation between average activities shown in (d) and various behaviors are shown. Blue and magenta plots cor-respond to gating and non-gating, respectively. In both type of networks, 10 inde-pendent simulations were performed. P-values were calculated by two-sided Welch's t-test.
