# Supplementary material for: Neural circuit mechanisms of hierarchical sequence learning tested on large-scale recording data
Source: PLoS Comput Biol. 2022 Jun 21;18(6):e1010214. doi: 10.1371/journal.pcbi.1010214 (PMC9249189; doi:10.1371/journal.pcbi.1010214)
Supplement: S9 Fig — (a) A schematic illustration of the neuron model with three dendritic compartments without gating. (b) Trained weight matrices are displayed for afferent inputs to three dendritic branches of three example neurons. (c) (d) Somatic and dendritic activities of the three neurons in (b) are shown. (PDF) [file pcbi.1010214.s009.pdf]

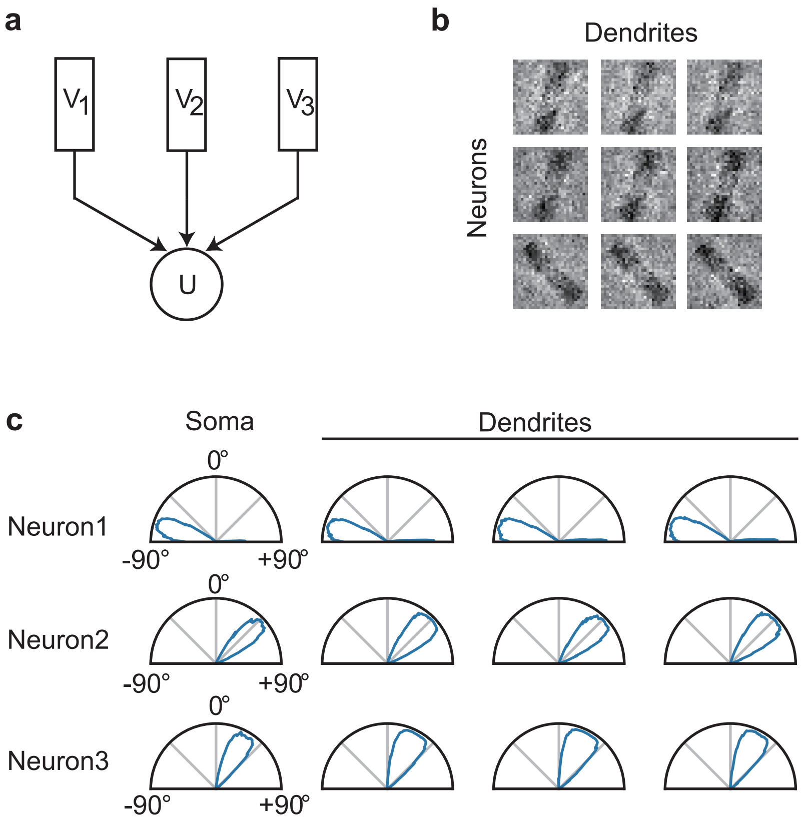

S9 Fig. Multi-dendrite neuron model without gating. (a) A schematic illustration of the neuron model with three dendritic compartments without gating. (b) Trained weight matrices are displayed for afferent inputs to three dendritic branches of three exam-ple neurons. (c) (d) Somatic and dendritic activities of the three neurons in (b) are shown.
