# Supplementary material for: Neural circuit mechanisms of hierarchical sequence learning tested on large-scale recording data
Source: PLoS Comput Biol. 2022 Jun 21;18(6):e1010214. doi: 10.1371/journal.pcbi.1010214 (PMC9249189; doi:10.1371/journal.pcbi.1010214)
Supplement: S10 Fig — Dendrites received additive recurrent inputs as well as afferent inputs and the dendritic activity underwent recurrent gating. (a) As in Fig 2, the trained network segmented two overlapping chunks in the presence of additive recurrent inputs. (b) Normalized average responses of two emergent assemblies during the presentations of chunk 1 and chunk 2. (c) PCA showed that the different chunks were distinguishable by different low-dimensional trajectories, of which the black, orange, and blue portions indicate the periods of random spike input, chunk 1 and chunk 2, respectively. (PDF) [file pcbi.1010214.s010.pdf]

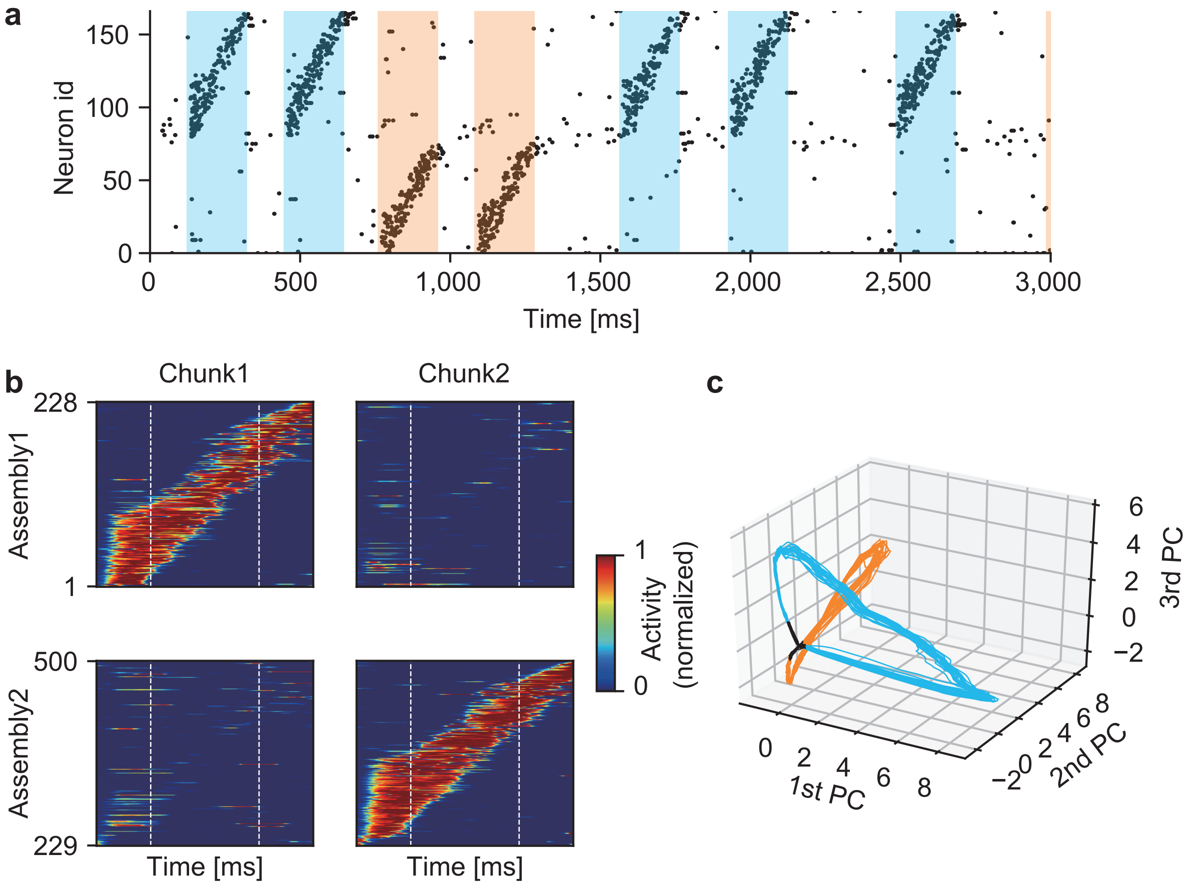

S10 Fig. Sequence learning in the copresence of recurrent gating and recurrent input. Dendrites received additive recurrent inputs as well as afferent inputs and the dendritic activity underwent recurrent gating. (a) As in Fig. 2, the trained network segmented two overlapping chunks in the presence of additive recurrent inputs. (b) Normalized average responses of two emergent assemblies during the presentations of chunk 1 and chunk 2. (c) PCA showed that the different chunks were distinguishable by different low-dimensional trajectories, of which the black, orange, and blue portions indicate the periods of random spike input, chunk 1 and chunk 2, respectively.
