# Supplementary material for: Neural circuit mechanisms of hierarchical sequence learning tested on large-scale recording data
Source: PLoS Comput Biol. 2022 Jun 21;18(6):e1010214. doi: 10.1371/journal.pcbi.1010214 (PMC9249189; doi:10.1371/journal.pcbi.1010214)
Supplement: Attachment — Submitted filename: Asabuki_etal_PlosCB_2021_review.pdf [file pcbi.1010214.s011.pdf]

In this paper, the authors built on their previous modeling work and showed that a recurrent spiking network with dendrites can develop neural responses selective for different temporally-extended input spike patterns, even though these patterns share same elements. The network does so by 1) having a recurrent multiplicative gating mechanism that scales the current from dendrite to soma by a gating factor determined by the instantaneous activity of the network, and 2) being trained on an unsupervised learning algorithm that maximizes the consistency between the (gated) dendritic and the somatic activities of each neuron. The authors argue that these two elements are crucial for the ability of the network to perform the temporal pattern segmentation task. I think the paper presents an interesting idea, but would benefit from a more detailed dissection of the mechanism that enables the network to perform the task, including a more rigorous exploration about the necessary model ingredients for performing the task as well as the robustness of its performance to parameter variations.

<u>Major comments:</u>

- Why can, and how does, the model perform the tasks?

    - It is not clear to me the connection between maximizing the somato-dendritic consistency and the network's ability to perform the segmentation task. I think the paper would benefit greatly from a more detailed dissection into the network mechanism that enables the network to perform this task, and how this mechanism emerges as a requirement for the training objective.

      First of all, the authors should emphasis in the paper that it is the consistency between the somatic activity and the **gated** dendritic activity that is being maximized (Equation 14, 15), not the consistency between the actual somatic and dendritic activity.

      Then, it is still not clear to me the connection between this training objective and the network's ability to perform the task. For example, it seems the loss function (Equation 14) would always be low, since both U and V* are controlled by the gating factor c. I think as a starting point it might be useful to see the somato-dendritic consistency as a function of training steps.

      Finally, what features in the trained network enable it to perform the segmentation task? I will leave it up to the authors to decide how exactly to go about exploring this, but I would think the recurrent weight matrix that targets the gate (w^{net(c)}) may be useful to look at.

    - This is related to the previous question on the neural mechanism. The neurons in the network show sequential firing that can rescale and even reverse with the duration of the input spike pattern. This is pretty interesting. I also noticed that the neurons in the previous work (Asabuki and Fukai, Nature Communication 2020) show homogeneous and sustained firing. Can the authors show what circuit mechanism underlies this scalable and reversible sequential firing in the current model? Could it be because of the short time constant of the somatic membrane

potential such that each neuron is only reacting to the instantaneous input and barely integrates inputs over time?

- The authors argued for a division of labor between multiplicative and additive recurrent interactions, where the former is necessary for segmenting different input patterns where the latter is responsible for pattern completion. They also showed in Supplementary Figure 1 that a recurrent network with only additive recurrent interactions fails to segment sequence "CED" from "AEB". However, it is not clear to me that multiplicative gating is really a necessary condition for performing the segmentation task, since both types of connections serve the purpose of self-amplification. In addition, it seems totally possible that a vanilla RNN used in machine learning can perform the task in Fig.S1 when trained with backpropagation. Therefore, I wonder if the authors can give some intuitions as to why multiplicative gating is necessary, or beneficial, for the tasks they are considering.

- The model has many free parameters. Therefore, I think the paper would benefit from a more thorough exploration of the robustness of the model performance to variations model parameters.

<u>Minor comments:</u>

- Figure 2: I wonder why in the sequence "AEB", the syllable E is longer than A and B?

- Figure 3d: Could the author explain what mechanism causes the time courses of the three traces? It seems that the raise of the gating factors all lag behind the corresponding raise of the dendritic activity (Figure 3c), but only the orange trace is able to reach some threshold to cause the somatic firing.

- Similarly, could the author expand on what they mean by "memory effect" on line 164?

- Line 179: it is hard to tell that the overlapping segment of the trajectories in Figure 4b middle and Figure 4c-e represent the shared pattern E. I think more explanation is needed for those panels.

- Figure 6d-g: it is interesting that the authors found repetition of the same neural activity pattern from the large-scale neuropixel recording. I wonder whether there is any way to connect this finding back to the experimental data. For example, do those neural patterns correspond to stereotypical movement patterns of the animal?
- Figure 6h: how was the simulation done when the number of input neuron is larger than 6532? Are some of the neural data synthetic? It would also be good to add a legend to show which curve corresponds to which condition.

- Line 251 and Fig.S4: Could the author quantify to what extent the cell-assembly structures detected by the previous model are vague?

- Figure 7: I think more information should be provided on the simulation. For example, what dataset is the model trained on?

- Methods, Equation (4): could the authors discuss the biological plausibility of a multiplicative (rather than additive) gating term \lambda?

- Methods, Equations (5-6): could the authors explain the necessity of these normalization steps. Would using c and V, rather than chat and Vhat, lead to problems?

- Methods, Equations (12): the input in the RHS of Equation 12 is scaled by 1/tau, but I am not sure what the biological interpretation of this is.

- Methods, Equations (15): what is g_L?

- Methods, Equation (17): it seems to me that the term in the middle, \phi(V_i*) should be replaced by \beta? The authors might want to check the derivation in Equations (16-17).

- Methods, Equation (23): … (-U_{i,k}(t) + Vhat_{i,k}(t)) – should U_{i,k} be U_{i} here?

- Methods, Line 510-511 (Figure 6E): how did the authors determine how many groups to have in the first place?
