# Supplementary material for: Neural circuit mechanisms of hierarchical sequence learning tested on large-scale recording data
Source: PLoS Comput Biol. 2022 Jun 21;18(6):e1010214. doi: 10.1371/journal.pcbi.1010214 (PMC9249189; doi:10.1371/journal.pcbi.1010214)
Supplement: Attachment — Submitted filename: point-to-point-reply.docx [file pcbi.1010214.s012.docx]

Point-to-point reply

Ms #: PCOMPBIOL-D-21-01729

We thank all reviewers for their prompt and valuable feedback to our manuscript. To meet the criticisms by the reviewers, we have included results of additional simulations and novel analyses in the revised manuscript. The major revisions made in the manuscript are: We have modified/included 9 novel figures (Figures 1a-b, 4b-d, and 5d and Supplementary Figures S1, S4, S5, S7, S8 d-e, and S9). Below, we list our point-to-point reply to the reviewers' comments. Slanted fonts indicate the reviewers’ comments and normal fonts our reply.

Reviewer #1

*Major comments:*

*First of all, the authors should emphasis in the paper that it is the consistency between the somatic activity and the gated dendritic activity that is being maximized (Equation 14, 15), not the consistency between the actual somatic and dendritic activity.*

**Reply –** We agree with the reviewer that this point should be emphasized. We have included the sentence in ll. 119-122 of the revised manuscript.

*Then, it is still not clear to me the connection between this training objective and the network’s ability to perform the task. For example, it seems the loss function (Equation 14) would always be low, since both U and V* are controlled by the gating factor c. I think as a starting point it might be useful to see the somato- dendritic consistency as a function of training steps.*

**Reply –** We have included the results of the simulation suggested by the reviewer. In S1 Fig, the learning period was divided into multiple sections, and the correlation coefficient between somatic and dendrite activity was calculated in each section. As expected, the correlation increases as learning progresses. We have mentioned this behavior in the manuscript in ll.138-139.

*Finally, what features in the trained network enable it to perform the segmentation task? I will leave it up to the authors to decide how exactly to go about exploring this, but I would think the recurrent weight matrix that targets the gate (w^{net(c)}) may be useful to look at.*

**Reply –** The reviewer raised an important question. We analyzed the structure of recurrent weight matrix that targets gate (w^{net(c)}) of trained network in S4 Fig. In summary, we found that the structure of trained recurrent gating connections supports a sequential response of each assembly. Further, the connections showed strong suppression between assemblies that correspond to the same pattern in different contexts. These features in the trained network enable the context-dependent sequence segmentation. We have explained the results in ll.178-192 in the revised manuscript.

*o This is related to the previous question on the neural mechanism. The neurons in the network show sequential firing that can rescale and even reverse with the duration of the input spike pattern. This is pretty interesting. I also noticed that the neurons in the previous work (Asabuki and Fukai, Nature Communication 2020) show homogeneous and sustained firing. Can the authors show what circuit mechanism underlies this scalable and reversible sequential firing in the current model? Could it be because of the short time constant of the somatic membrane potential such that each neuron is only reacting to the instantaneous input and barely integrates inputs over time?*

**Reply –** The reviewer’s speculation is correct. Our time constant of neuron is 15ms, which is much shorter than the 200ms duration of sequences. This shorter time constant should make the neural response sensitive to instantaneous stimulus. Also, unlike previous model of which the recurrent inhibitory connections are plastic, we assumed static inhibitory connections in the current model. Such fixed inhibition leads to competition among model neurons, hence makes the activation sparser.

*o The authors argued for a division of labor between multiplicative and additive recurrent interactions, where the former is necessary for segmenting different input patterns where the latter is responsible for pattern completion. They also showed in Supplementary Figure 1 that a recurrent network with only additive recurrent interactions fails to segment sequence “CED” from “AEB”. However, it is not clear to me that multiplicative gating is really a necessary condition for performing the segmentation task, since both types of connections serve the purpose of self-amplification. In addition, it seems totally possible that a vanilla RNN used in machine learning can perform the task in Fig.S1 when trained with backpropagation. Therefore, I wonder if the authors can give some intuitions as to why multiplicative gating is necessary, or beneficial, for the tasks they are considering.*

**Reply –** As the reviewer mentioned, various types of recurrent networks are powerful tools for sequence learning. However, to our knowledge, most such networks are trained in a supervised manner, unlike our proposed model that is trained to maximize the consistency between compartments of neurons. Although we found that our gating recurrent network is able to perform complex sequence segmentation tasks like Long Short-Term Memory (LSTM), the underlying mechanism of how our gating works in the principle of consistency maximization is still an open question. We have mentioned in ll.422-424 as “It is intriguing to ask whether and how recurrent networks learn an optimal forget gate for unsupervised learning of hierarchical sequences in addition to the proposed gate”.

*o The model has many free parameters. Therefore, I think the paper would benefit from a more thorough exploration of the robustness of the model performance to variations model parameters.*

**Reply –** Although we have many free parameters (e.g., learning rate), we thought the parameter $\gamma$ and the strength of inhibition are most critical as these affect the model dynamics directly.

In S5b Fig and S5c Fig, we have included the simulation results testing robustness of model performance to various model parameters. In our learning rule, the standardization process in eqs. (5)-(6) is crucial to avoid trivial solution (please see the reply below). As standardization in both the dendritic and gating activations depends on a single parameter $\gamma$, we first explore the model robustness against this parameter. We found that performance of learning decreased as the parameter $\gamma$ increased. This result is reasonable as larger value of $\gamma$ leads to shorter period for calculation of moments of potential, making the standardization unstable. The second parameter we tested is a strength of static recurrent inhibitory connections. We found the model shows best performance when the strength of inhibition is moderate. As the peaks of performance changes are not steep, our model choice was not fine tuning. We have explained the results in ll.193-208 in the revised manuscript.

*Minor comments:*

*o Figure 2: I wonder why in the sequence “AEB”, the syllable E is longer than A and B?*

**Reply –** Here, we wanted to show the model works in a difficult setting of task. Intuitively, learning becomes difficult by increasing the duration of shared syllable. Therefore, we thought longer duration of syllable E would be appropriate as a first demonstration of network performance.

*o Figure 3d: Could the author explain what mechanism causes the time courses of the three traces? It seems that the raise of the gating factors all lag behind the corresponding raise of the dendritic activity (Figure 3c), but only the orange trace is able to reach some threshold to cause the somatic firing.*

**Reply –** Differences in the time course of dendrites and gating activities are due to differences in input. Here, all dendrites receive afferent inputs, leading rapid response to the external stimuli. Gating factors, on the other hand, are driven by recurrent inputs from network neurons and therefore always contains a delay. Further, somatic firing occurs only by coincident activation of dendritic and gating factors. In Fig. 3c-e, only orange traces in dendrite and gating factor show temporal coincidence, hence leads to somatic response. All other dendritic activities are suppressed in soma due to the low value of gating activations.

*o Similarly, could the author expand on what they mean by “memory effect” on line 164?*

**Reply –** We wanted to state that as our gating is determined by recurrent input, which depends on the network state at previous timestep, it has some memory effect. We modified the statement in the revised manuscript as “This selective gating is thought to arise from a memory effect generated by recurrent synaptic input, which is determined by previous state of a network.” in ll.171-173.

*o Line 179: it is hard to tell that the overlapping segment of the trajectories in Figure 4b middle and Figure 4c-e represent the shared pattern E. I think more explanation is needed for those panels.*

**Reply –** We agree with the reviewer's suggestion. We clarified the part of trajectories not separated by presentation of shared pattern E by including arrows in the figure. We explained the behavior of trajectories in ll.217-221 in the manuscript.

*o Figure 6d-g: it is interesting that the authors found repetition of the same neural activity pattern from the large-scale neuropixel recording. I wonder whether there is any way to connect this finding back to the experimental data. For example, do those neural patterns correspond to stereotypical movement patterns of the animal?*

**Reply –** Unfortunately, it is still an open question what features in a behavior data are encoded in each of the detected assemblies. Here, we only show the same behavior is encoded by repetition of multiple assemblies. However, we found the activity of the gated network show better prediction of behavior data than the non-gating model (please see the reply below).

*o Figure 6h: how was the simulation done when the number of input neuron is larger than 6532? Are some of the neural data synthetic? It would also be good to add a legend to show which curve corresponds to which condition.*

**Reply –** Here, we changed the number of input neurons 1, 2/3, 1/3 times greater than original 6,532 neurons. Therefore, we did not consider the situation where the number of neurons increases from the original number. We also included legends in Fig.6.

*o Line 251 and Fig.S4: Could the author quantify to what extent the cell-assembly structures detected by the previous model are vague?*

**Reply –** We asked to what extent the population averaged activity of trained network in two scenario (i.e., with gating or no gating) correlates to the behavior. We found the population averaged activity of trained model with gating shows higher value of correlation than the previous non-gating model to behaviors (S8e Fig in revised manuscript). Although we did not evaluate the quality of clustered structure of network neurons, these results suggest the proposed model explains the behavior data better than previous model. We modified the statement in the revised manuscript in ll.309-313.

*o Figure 7: I think more information should be provided on the simulation. For example, what dataset is the model trained on?*

**Reply –** We thank the reviewer for this suggestion. We have provided the detailed information on the simulation protocol in ll.328-333.

*o Methods, Equation (4): could the authors discuss the biological plausibility of a multiplicative (rather than additive) gating term \lambda?*

**Reply –** Although previous experimental finding has shown that postsynaptic inhibition affects the membrane potential in a form of multiplication, suggesting the implementation of multiplication within neurons, our gating mechanism could be interpreted in much broader sense. As no experimental evidence has yet been found that recurrent inputs gate the afferent inputs, how our gating mechanism could be implemented within neuron is an open question. We have mentioned the above discussion in ll.404-407 of revised manuscript.

*o Methods, Equations (5-6): could the authors explain the necessity of these normalization steps. Would using c and V, rather than chat and Vhat, lead to problems?*

**Reply –** Our learning rule seeks to minimize the error between gated dendritic and somatic activities, which itself leads to a trivial fixed point. As proposed in our previous work, the standardization enable the model to avoid a trivial solution to the learning rule, and therefore ensure successful learning of nontrivial temporal features. We have stated the above in a revised manuscript in ll.485-487.

*o Methods, Equations (12): the input in the RHS of Equation 12 is scaled by 1/tau, but I am not sure what the biological interpretation of this is.*

**Reply –** Equations 12-13 follows the formula of double exponential kernel. Here, variable X^ext in eq.12 is a spike train represented as a summation of Delta function as in eq.11. Each Delta function corresponds to the electric charge that each spike transmits. The scaling with 1/tau ensures that the last term in eq.12 is in unit of current. We have explained the above point in ll. 503-504 in the revised manuscript.

*o Methods, Equations (15): what is g_L?*

**Reply –** We included the explanation of *g_L* in ll.518.

*o Methods, Equation (17): it seems to me that the term in the middle, \phi(V_i*) should be*

*replaced by \beta? The authors might want to check the derivation in Equations (16-17).*

**Reply –** We thank the reviewer her/his careful review. We modified the notation.

*o Methods, Equation (23): ... (-U_{i,k}(t) + Vhat_{i,k}(t)) – should U_{i,k} be U_{i} here?*

**Reply –** We thank the reviewer her/his careful review. We modified the notation.

*o Methods, Line 510-511 (Figure 6E): how did the authors determine how many groups to have in the first place?*

**Reply –** The number of groups was not preconfigured. We grouped neurons such that all pairs in a group had a correlation coefficient greater than 0.2.

Reviewer #2

*Major comments:*

*I felt it would be nice to have a sub-section dedicated to the presentation of the model at the beginning of the result section. For now, the presentation of the model is mixed with the first results of the paper, I have found it makes things a bit confusing. And I also felt it would be nice to have a high-level description of the learning procedure, which is not present in the main text if I am correct. In particular, it would be nice to explain how they extended their previous learning algorithm for it to deal with recurrent connections, and say a bit more of the relationship between the formula they derive and the online learning rule they introduce (cf method section). It would also be nice to be able to have, somewhere, a sense of the hyper-parameters that have to be tuned to fit this model.*

**Reply –** We would like to thank the reviewer for her/his comments that helped to make the description in the manuscript clearer. We separated the subsection of model explanation and the demonstration of our first task. Further, in ll. 119-125 of the revised manuscript, we emphasized that learning rule for both afferent and recurrent gating connection were derived to minimize the prediction error between somatic and gated dendritic activity and described the differences of learning rules for afferent and recurrent gating connections. We also demonstrated the performance dependences on various parameters in ll.193-208 of the revised manuscript (please see the reply below).

*I have found Fig. 2, 3 and 4 very compelling. Related to the above comment about hyper-parameters, it would be nice to show how these hyper-parameters have been varied before presenting the control result of Fig.S1, that shows that recurrent gating is crucial for context-dependent segmentation.*

**Reply –** Although we have many free parameters (e.g., learning rate), we thought the parameter $\gamma$ and the strength of inhibition are most critical as these affect the model dynamics directly.

In S5 Fig, we included the simulation results testing robustness of model performance to various model parameters. In our learning rule, the standardization process in eqs. (5)-(6) is crucial to avoid trivial solution (please see the reply below). As standardization in both the dendritic and gating activations depends on a single parameter $\gamma$, we first explore the model robustness against this parameter (S5b Fig). We found that performance of learning decreased as the parameter $\gamma$ increased. This result is reasonable as larger value of $\gamma$ leads to shorter period for calculation of moments of potential, making the standardization unstable. The second parameter we tested is a strength of static recurrent inhibitory connections (S5c Fig). We found the model shows best performance when the strength of inhibition is moderate. As the peaks of performance changes are not steep, our model choice was not fine tuning. We have explained these results in ll.193-208 of the revised manuscript.

*In Fig.5, the author, if I have understood correctly, want to probe how robust is the learning with respect to variation in the overall temporal scale of the inputs. I would like to see how temporally scaling the input sequence of Fig.3 would affect the down-stream (If I have understood, here the authors introduce another type of input sequence). It would be nice to clearly state in the main text how much warping can be tolerated before neural activity in the model loses its nice context-dependent property. Having such a limit on the degree of acceptable time warping would help better understand the mechanism proposed here as well as its potential limitations. The rationale behind using rank-order correlations could be expanded, for readers not familiar with this measure. Also I did not get what panel e. is about.*

**Reply –** The reviewer raised an interesting question. For the input spike pattern used in the task in Fig. 3, we explored how much warping can be tolerated before the model loses its context-dependent property. We measured the performance of both gating and non-gating model against various sequences with different relative duration (RD). As shown in Fig.5d, when the RD was 3 or less, the gating model showed higher performance than non-gating model, but there was no clear difference between the two when RD increased further. From this result, our gating model, which enables context-dependent computations, was able to learn up to RD=3 stretching in this task. We have added a description of this result in the manuscript in ll. 248-254.

Also, we have mentioned the motivation of using rank-order correlation in ll.241-245 of revised manuscript. Regarding panel e (panel f in the revised Fig. 5), the figure shows the network trained with only the original pattern (top panel of revised Fig. 5e) respond to the novel time-reversed pattern (bottom panel of revised Fig. 5e) in a reverse order. We have emphasized in ll. 260-263 of revised manuscript that only original pattern was used during training and the reverse pattern was novel in this case.

*In Fig.6, the authors apply their methodology on calcium imaging data. I have been quite confused by this section. In particular, while the previous section states that the mechanism is useful for context-dependent computations, here I did not get whether the behavior of the animals show any form of context-dependencies. Also, it would be nice for readers to understand, how hyper-parameters (e.g. number of neurons in the model) are chosen, and how they impact the result of the analysis.  As for the first data-set, I have understood that the methodology allows to order recorded neurons so as to exhibit nice sequential activations. It seems the procedure they use to get there is quite intricate, aren’t there more simple ways to do so ? Is the gating mechanism crucial here ? As for the second dataset, the presentation of the data is too succinct. For instance to understand what those 500ms correspond to (e.g. is it the whole dataset or a subset chosen as an example ; what is the behavior, besides pupil dilatation, during those 500ms ? ; what is the distinction, in terms of behavior, between the early and late epochs). Here the authors claim that their new method is better than the previous one at exhibiting a form of context dependency (early VS late epoch), but comparing, by eye, figures S4.c and 6.f does not lead to the conclusion of this paragraph. It would be nice to have something more quantitative to substantiate the claim regarding the superiority of the new method.*

**Reply –** We thank the reviewer for important suggestion. As the reviewer suggested, we have included the detail of dataset in ll.282-285 together with novel figure (S7 Fig) to demonstrate the behaviors of mice. Although our network found structural changes between early and late phase of activation in visual cortex data, behaviors did not show clear distinction. We mentioned this in ll. 293-296 in the revised manuscript. Further, we have included new paragraph for explaining the role of gating mechanism in the analysis of neural data in ll.302-314 of the revised manuscript. In the analysis of hippocampal CA1, model without gating shows no clear separation of sequences correspond to forward and backward run (S8a Fig), while separated in recurrent gating model (Fig.6b). Further, in the analysis of visual cortex data, we found the population averaged activity of trained model with gating shows higher value of correlation than the previous non-gating model to behaviors (S8e Fig), indicating the superiority of proposed model.

*In Fig.7, the authors want to discuss recent experimental comparisons of « dendritic receptive fields » VS « somatic receptive fields ». The introduction of the corresponding section could be expanded to more clearly state the rationale behind this section. Indeed it can be confusing given the fact that the main message of the paper is centered on the idea of hierarchical sequence, while here there are no notion of hierarchy on the input sequences. Again it would be nice to see whether a model with three dendritic branches, without gating would lead to the same results or not.  It would also be nice to explain how the learning procedure has to be adapted when adding multiple compartments.*

**Reply –**In S9 Fig, we checked the model behavior with three dendritic branches, without gating. We found in such model without gating, all three dendritic branches show identical preferred stimulus orientation. This result is reasonable because non-gating model lacks a competition among branches. Also, the synaptic learning rules for afferent and gating recurrent connections in the case of multiple dendrites are described in eqs. (26) and (27) in Methods.

*As a side note, I was wondering whether having a model with two branches to fit the hippocampal data for animals on linear track (Fig.6) could give interesting results.*

**Reply –** We thank the reviewer for interesting suggestion. However, this was not easy to investigate in this revision as the computation time increases in multiple dendrites. We wish to leave this interesting problem for a future work.

*The last part about the role of non-gating recurrent connections was clear and interesting. I again had the same type of comments: would it be possible to describe in the main text how the learning procedure is able to deal with this new connections compared to the gating ones.*

**Reply –** Here, non-gating recurrent connections were trained with the same rule as for afferent connections. We emphasized this in ll.347-348.

Reviewer #3

*Fig 1: The circuit diagram could be more informative. In particular there is a gap in detail between the Fig 1a and 1b and the actual equations (1)-(11); for example, where is the “local potential” c? What is its biological meaning? None of the weights or incoming connections are noted on the schematic.*

**Reply –** We thank the reviewer for her/his valuable suggestion. We modified Fig. 1 as follows. In Fig1.a, we included the schematic of dendritic compartments to emphasize each neuron is two-compartment. In Fig1.b, we included the label of each connection and the “local potential” c. We also modified Fig1.c to demonstrate each case of lambda leads to strong or weak attenuation.

Although previous experimental finding has shown that postsynaptic inhibition affects the membrane potential in a form of multiplication, suggesting the implementation of multiplication within neurons, our gating mechanism could be interpreted in much broader sense. As no experimental evidence has yet been found that recurrent inputs gate the afferent inputs, how our gating mechanism could be implemented within neuron is an open question. We have mentioned the above discussion in ll. 404-407 of revised manuscript.

*Fig 6: I found the comparison between this and the results without recurrent gating (S4) vague. What do you mean “the cell assembly structures were vague”?
Do you mean they overlap (in cell identities)? From Fig S4a, it does appear that different neurons are active during the forward and backward runs.*

**Reply –** We agree with the reviewer that we should evaluate the performance quantitatively. We asked to what extent the population averaged activity of trained network in two scenario (i.e., with gating or no gating) correlates to the behavior. We found that the population activity trained with gating shows higher value of correlation than the previous non-gating model against behaviors. Although we did not evaluate the quality of clustered structure of network neurons, these results suggest the proposed model explains the behavior data better than previous model. We included novel figures (S7 Fig and S8e Fig) and modified the statement in the revised manuscript in ll. 309-314.

*pg. 17,18: Define “softmax”*

**Reply –** We defined the “softmax function” in ll. 550, before showing the definition of recurrent gating in multi-dendrite case.

*The finding that the network learn assemblies to respond to complex sequences (e.g. ABCD vs. DCBA) is very interesting as is the mechanism for accomplishing this (dendritic activation + gating factors to distinguish between orderings). My question is what is the capacity of the network? i.e. how many such sequences can it learn, and how does this relate to the size of the network?
Can you make a prediction based on the network architecture?*

**Reply –** We have included novel S5a Fig to meet the reviewer’s question. We considered the network of sizes 300 and 600. For each of the network, learning was performed with the number of sequences of 3, 5, and 7. For the input sequences, we randomly selected sequences from 4! = 24 permutations of a set (a, b, c, d). After training, we evaluated the feature selectivity as a model performance. As expected, for both cases of network size, the performance decreases as the number of sequences increases. Although we did not find the clear capacity of networks, the performance of network size 600 was higher in all sequences than when it was 300. We have explained the result in ll.193-197 in the revised manuscript.

*Methods, pg 18: why is there a need for a “standardized potential”; i.e. \hat{c} and \hat{V}? Can you explain these quantities.*

**Reply –** Our learning rule seeks to minimize the error between gated dendritic and somatic activities, which itself leads to a trivial fixed point w = 0 of Eqs. (20)-(21). As proposed in our previous work [19], the standardization enable the model to avoid a trivial solution to the learning rule, and therefore ensure successful learning of nontrivial temporal features. We have stated the above in a revised manuscript in ll.485-487.
