# Supplementary material for: Neural circuit mechanisms of hierarchical sequence learning tested on large-scale recording data
Source: PLoS Comput Biol. 2022 Jun 21;18(6):e1010214. doi: 10.1371/journal.pcbi.1010214 (PMC9249189; doi:10.1371/journal.pcbi.1010214)
Supplement: Attachment — Submitted filename: point-to-point-_2nd.docx [file pcbi.1010214.s013.docx]

Point-to-point reply

Ms #: PCOMPBIOL-D-21-01729

*Reviewer #1: The authors’ additional analysis strengthens the paper and clarifies some of my confusions, but I still have a few outstanding questions:*

**Reply** –We thank the reviewer for her/his valuable feedback to our manuscript. To meet the criticisms by the reviewer, we have included additional description suggested by the reviewer in the revised manuscript. Below, we list our point-to-point reply to the reviewers’ comments. Slanted fonts indicate the reviewers’ comments and bold fonts our reply.

*- It is nice to see the structure of the connectivity matrix in Fig.S4, but it still confuses me how the learning objective, which is to maximize the consistency between the somatic and the gated dendritic potentials, relates to the development of this connectivity structure. I raised this question in my previous review as well, and the authors showed in Fig.S1 that the consistency increases during training. This is nice to confirm, but I think it would really strengthen the paper if the author could further develop the connection between the learning objective and the emergence of sequence-selective cell populations as well as all the other cool properties of the model.*

**Reply** **– The single-cell model proposed previously [19] could not perfectly segment similar sequence patterns as shown in Figs. 2 and 3. Though the consistency between somatic and dendritic activities forces the neuron to respond to a specific input pattern, it could not perfectly discriminate similar patterns involving overlapping components. In the recurrent gating network, recurrent input on each neuron selectively passes one of similar input patterns from the dendrite to the soma, enabling the context-dependent segmentation. This cooperative function of recurrent synaptic input is difficult to prove analytically but is understandable because both afferent and recurrent synapses are trained by the same learning rule to achieve the same goal, that is, an optimally predictable somatic response. We have added the above discussion in ll.195-203 of the revised manuscript.**

**We consider that an analytic proof is not so easy since a naive meanfield approach does not work. To see this, we simulated a single neuron gated by its own output through a** **self-recurrent synaptic connection. The self-gated neuron received synaptic inputs involving two chunks sharing their components (i.e., "abcd" and "dcba"). The typical responses shown below indicate that this neuron failed to learn a selective response to one of the chunks. The neuron responded to some components of the chunks irrespective of which chunk is presented.**

**These results suggest that clarifying the mechanism of recurrent gating requires a network-wide analysis beyond the meanfield-like view. Indeed, in many high-performance models of sequence processing, such as LSTM, a rigorous analysis of the underlying mechanisms is very hard. We wish to leave this difficult problem for future work.**

*- In figure S4 the authors showed the connectivity matrix W^c., which is helpful to understand the mechanism. Could the authors show the structure of W^v as well? It seems to me that given the right structure in W^c, W^v is not very important for this model to work, since the critical point is the coincidence of the external input and the gating factor c. Is this intuition correct?
Related, could the authors discuss the biological substrates of W^c and W^v? They are both projections from the soma to the neighboring dendrites, so it is not clear to me what might be the difference between their neural implementations.*

**Reply – We didn’t include additive recurrent connections W^v in the simulations shown in Fig. S4, meaning that the model had only afferent connections W^c. Therefore, as the reviewer correctly pointed out, W^v is unimportant for sequence segmentation. As we discussed in Fig. 8, the major role of W^v, when it exists, is to drive pattern completion in the absence of external stimuli (as in the conventional attractor networks). However, sequence segmentation per se does not rely on W^v.**

***W^v* is thought to represent recurrent synaptic connections projecting onto the dendrites of pyramidal neurons, but evidence for the gating recurrent connections *W^c* is currently not available. However, A recent study suggests that the thalamocortical feedback is necessary for reliable propagation of a synaptically evoked dendritic depolarization to the soma in layer-5 pyramidal neurons (Suzuki and Larkum (2020), ref.35). This phenomenon is reminiscent of the gating mechanism proposed in this study. Thus, such a mechanism may be realized on a large spatial scale by cortico-thalamocortical recurrent circuits. However, the biological mechanisms of recurrent gating is open to future studies. We have included the discussion described above at ll. 418-423 in the revised manuscript.**

*- I still did not understand the argument that standardizing V and c lets the system avoid a trivial fixed point. I could not find the relevant text in ref.19 except the following sentence "However, as shown previously, the online modifications given in Eqs. (4)–(7) prevent the function \psi^{som}_i(u_i(t)) from coinciding with \psi^{dend}(v*_i(t)). This in turn prevents a trivial fixed point w= 0 of Eq. (16)". Could the authors elaborate in more detail the necessity of standardizing V and c, or consequences of not doing so?*

**Reply** **–Our learning rule minimizes the time-averaged error between the somatic activity and (gated) dendritic activity. As the somatic activity is driven by the dendritic activity, consistency between the two activity patterns is improved as learning proceeds. Without standardization, our learning rule can minimize the error to zero by making both activities simultaneously zero (see Eq. 18). This trivial solution occurs when all synaptic weights vanish after learning. The standardization prevents the trivial solution by maintaining temporal fluctuations of O(1) in the somatic membrane potential, thus ensuring successful learning of nontrivial temporal features. Similarly, standardization was used to avoid a trivial solution in Slow Feature Analysis (Wiskott and Sejnowski, 2002). We have included the above arguments in ll.502-507 of the revised manuscript.**

*Some other minor comments:
- Could the authors specify in more details how the training is done? Are the patterns presented continuously or separated by a long delay (so that network is at baseline when each pattern is presented), or it does not matter (since the neuronal time constant is only 15 ms)?*

**Reply –In the previous manuscript, the input protocol used was only stated in the legend of Fig.2. In the revised manuscript, we have added the following statement to ll.137-139 of the main text: “Throughout this paper, we fixed the average firing rate of each input neuron at 5 Hz over the entire period of simulations, and chunks were separated by random spike trains with variable lengths of 50 to 400 ms.”**

*- For figure 6g, I am afraid I still did not understand the authors' response. Are the number of neurons for the three curves 6532*(1+1), 6532*(1+2/3) and 6532*(1+1/3)? If so, since only 6532 neurons are in the dataset, how did the authors determine the activity of the rest of the neurons?*

**Reply** –**In Fig.6g, we decreased rather than increased the number of neurons by multiplying factors smaller than 1 as 6532*1, 6532*(2/3) and 6532*(1/3). Therefore, the number of neurons considered here never exceed the original number 6532. We thought that the confusion occurred as we used the word "greater" instead of "smaller" in the legend of Fig. 6h. We have corrected the word.**

*- In figure 3d and S3c, why do the gating factors for the irrelevant sequence (e.g. orange and green in figure S3c upper panel) rise up during seemingly random periods within a trial?*

**Reply** **–Because the somatic response is regulated multiplicatively by the produce of dendritic activity and gated factor, the latter can show some arbitrariness in the trained model as far as the behavior of the gating factor does not spoil the minimization of the error between the somatic response and the (gated) dendritic activity. We hope that this explanation clarifies the reviewer's doubt.**

*- Line 347-348: “Non-gating recurrent connections were trained with the same rule as for afferent connections.” It would be more clear to write out the update equations explicitly in the Methods.*

**Reply** – **In Eqs. (16) and (20), we have replaced** ${\mathbf{w}_{\boldsymbol{i}}}^{\mathbf{net, ext(V)}}$ **with** ${\mathbf{w}_{\boldsymbol{i}}}^{\mathbf{net(V), ext(V)}}$ **to make the mathematical expression somewhat more explicit. Further, we have emphasized that the same learning rule was applied to the weights of afferent and additive recurrent inputs (ll. 542-543).**

*- Line 503: “\tau_s = 5 ms”. Does \tau_s refer to the \tau's in equations 12 and 13?*

**Reply – The parameter** $\boldsymbol{\tau}$ **in Eq. (13) is the membrane time constant defined below Eq. (4) and is different from** $\boldsymbol{\tau}$**_s_ in Eq. (12). We have briefly noted this fact below Eq. (13).**

*- Line 352-354: “Since only additive recurrent input, but not recurrent gating, can activate postsynaptic cells, the additive input generates a reverberating activity, which may in turn assist the retrieval of learned sequences.” Why can’t recurrent gating activate postsynaptic cells? In general, a network without additive recurrent connections would also be able to generate reverberating activity as well, is that correct?*

**Reply – Reverberating activity is generally generated and maintained by synaptic current that is reverberated in a network by (additive) recurrent connections. This is because activity evoked in a neuron by external stimuli never propagates to other neurons in the network without recurrent connections. The same is true in our model: the net driving current "I" flowing from the dendrite into the soma is "multiplicatively" gated by the gating factor "G(R)", which is regulated by recurrent synaptic input "R":** $\boldsymbol{I\propto G(R)D}$**. Even if G(R) is open (i.e., G(R)>0), I contains no driving forces from other neurons in the network if D does not have additive recurrent input. In Fig. 8, we studied pattern completion in the absence of external cues. In this situation, D is zero without additive recurrent input and hence the somatic responses also become zero irrespective of the values of G.**

*Reviewer #2: I thank the authors for the work they have produced to address the concerns I raised. I am fully satisfied with their answers, I think this manuscript is ready for publication in PLoS CB.*

**Reply** **– We thank the reviewer for her/his recommendation.**

*Reviewer #3: None*
